# A 10-Year Immunopersistence Study of Hepatitis E Antibodies in Rural Bangladesh

**DOI:** 10.1093/aje/kwy044

**Published:** 2018-03-23

**Authors:** Brittany L Kmush, Khalequ Zaman, Mohammed Yunus, Parimalendu Saha, Kenrad E Nelson, Alain B Labrique

**Affiliations:** 1 Department of International Health, Bloomberg School of Public Health, Johns Hopkins University, Baltimore, Maryland; 2 Infectious Disease Division, International Centre for Diarrhoeal Disease Research, Bangladesh, Dhaka, Bangladesh; 3 Department of Epidemiology, Bloomberg School of Public Health, Johns Hopkins University, Baltimore, Maryland

**Keywords:** antibodies, antibody persistence, hepatitis E virus, seroepidemiologic studies

## Abstract

Hepatitis E virus (HEV) is a major cause of acute viral hepatitis in Southeast Asia. Several studies have suggested that antibody persistence after HEV infection may be transient, possibly increasing the risk of reinfection and contributing to the frequency of outbreaks in HEV-endemic regions. The specific conditions under which antibodies to HEV are lost, or “seroreversion” occurs, are poorly understood. Here, 100 participants from population-based studies in rural Bangladesh were revisited in 2015, 10 years after a documented HEV infection, to examine long-term antibody persistence. Twenty percent (95% confidence interval: 12.0, 28.0) of the participants no longer had detectable antibodies at follow-up, suggesting that antibodies generally persist for at least a decade after infection in rural Bangladesh. Persons who were seronegative at follow-up were generally younger at infection than those who remained positive (14.4 years vs. 33.6 years; *P* < 0.0001). This age-dependent antibody loss could partially explain cross-sectional seroprevalence data from Southeast Asia, where children have reportedly low antibody prevalence. The results of this study provide new insight into the immunological persistence of HEV infection in a micronutrient-deficient rural population of South Asia, highlighting the importance of age at infection in the ability to produce long-lasting antibodies against HEV.

Hepatitis E virus (HEV) is a major cause of acute viral hepatitis and is responsible for at least 20 million infections every year in developing countries (1). Hepatitis E disease is usually a self-limiting illness similar in clinical presentation to hepatitis A (2). However, hepatitis E can be very severe in pregnant women, causing fulminant hepatic failure and death, with a 30% case fatality rate (compared with 1%–2% in the general population) (3, 4). The immunological profile of HEV is rather different from that of other viral hepatitis infections, as it does not seem to infect children at a young age, as evidenced by cross-sectional anti-HEV antibody prevalence surveys in which children under 10 years of age have very low antibody prevalence (5).

In Southeast Asia, HEV causes outbreaks almost every year, with the majority of disease cases being seen in adults. Usually, under poor sanitation conditions, children are exposed to many enteric pathogens at a young age. As is seen with a typical example, hepatitis A, during a first exposure children develop antibodies against the virus, which protects them from getting sick or getting infected with the virus in the future. In low-resource areas, such as Bangladesh, nearly 90% hepatitis A virus antibody prevalence is achieved by age 10 years (6). However, this pattern of infection and disease has not been observed with HEV, and several populations across South Asia experience large epidemics of hepatitis E almost every year, often resulting in thousands, if not tens of thousands, of adult cases, despite the likely frequent exposure to the virus (7–11). These epidemics imply that the human immune system responds differently to HEV. One possible explanation is that the antibodies produced in response to HEV infection are short-lived, thereby leaving hosts susceptible to future infections (12).

During an acute HEV infection, anti-HEV immunoglobulin M is usually detectable around the start of clinical illness, with concentrations markedly decreasing over the next 5 months (13). Levels of anti-HEV immunoglobulin G increase during the end of the acute phase of the illness and into the convalescent phase, and then decrease over time (14). Decreasing titers are not unique to HEV, with many infectious agents displaying a similar pattern (15). However, decreasing titers are probably particularly relevant to HEV due to the frequency of large outbreaks in endemic areas despite constant exposure to the virus. To date, studies examining the long-term persistence of antibodies after HEV infection have only followed small numbers of patients for a short period of time—generally a maximum of 5 years with fewer than 5 people, studied 8–12 years after infection (16–21). Rapidly waning and undetectable levels of antibodies may contribute to the frequent outbreaks seen throughout Southeast Asia.

HEV is considered a major cause of acute viral hepatitis in Bangladesh, even though only 3 HEV outbreaks have been recorded (22–25). Several population-based studies of HEV in rural Bangladesh have been completed over the past 10 years; investigators have found a 22.5% prevalence of anti-HEV antibodies and an incidence rate of 64 infections per 1,000 person-years (26, 27). The careful prospective follow-up of this population over the past decade afforded us a unique opportunity to revisit persons documented to have had incident, natural infections. Unlike prior studies of HEV immunopersistence, these cohorts reflect carefully documented infections which occurred under research conditions, outside of outbreak settings. We revisited persons from these previous studies to determine the duration of persistence of antibodies acquired in the course of natural infection. The development of a successful vaccine candidate has increased the need to understand the duration of persistence of antibodies and protection after HEV infection in order to implement the most cost-effective disease control strategies.

## METHODS

### Participant selection

Participants were selected from the Matlab Health and Demographic Surveillance System (MHDSS) cohort, compiled by the International Center for Diarrhoeal Disease Research, Bangladesh (ICDDR,B) in southern Bangladesh. Over 100,000 people living in 67 villages were included in the parent cohort, which was set up in the 1970s and has been continuously followed since.

**Figure 1.**
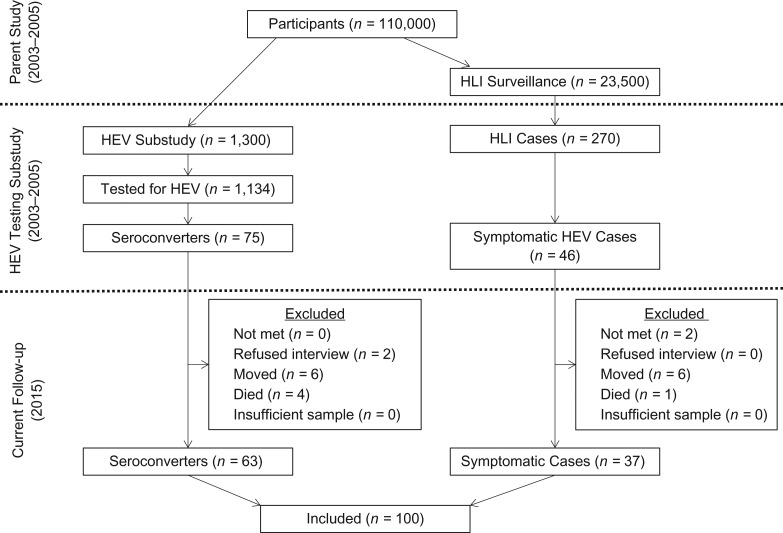
Cohort selection and follow-up of participants in a study of persistence of hepatitis E virus (HEV) antibodies, Matlab, Bangladesh, 2003–2015. HLI, hepatitis-like illness.

In 2003, 1,300 people were randomly selected from the MHDSS cohort to be included in a study of the epidemiology of HEV. All participants in the MHDSS cohort over 1 year of age were eligible for inclusion (26, 27). Of these 1,300 potential participants, 1,134 were enrolled in the study and were tested for HEV antibodies (26). Of the 879 who were seronegative at baseline, 75 seroconverted (became positive for antibodies to HEV, or anti-HEV-positive) during the 18 months of follow-up, indicating that they had experienced an asymptomatic HEV infection (Table 1, Figure 1) (27).

**Table 1.** Characteristics of Participants in a Study of the Persistence of Hepatitis E Virus Antibodies (*n* = 121), Matlab, Bangladesh, 2015

Participant Type	No. of Persons			Dates Studied		Total Time Since Exposure, years	Age at Enrollment, years		Clinical Characteristics at Enrollment
	Total	Men	Women	Enrollment	Follow-up		Mean	Range	
HEV seroconverter	75	30	45	2003–2005	2015	10–12	34	2–71	Asymptomatic HEV infection
Acute case of hepatitis E illness	46	31	15	2004–2006	2015	9–11	23	3–62	Symptomatic HEV infection first identified by symptoms, then by serological analysis

Abbreviation: HEV, hepatitis E virus.

In 2004, one block of the MHDSS cohort containing approximately 23,000 people was selected to undergo prospective surveillance for hepatitis-like illness (28). A total of 270 individuals were identified by community health research workers as having cases of hepatitis-like illness based on a symptom algorithm, including fever, anorexia, yellow skin or eyes, dark urine, and clay-colored stools (28). After symptom screening, patients were tested for a panel of hepatitis viruses, including HEV. Forty-six participants were found to be positive for acute hepatitis E illness (Table 1, Figure 1) (28).

In 2015, the 121 cases of hepatitis E identified in the above studies (75 asymptomatic cases and 46 symptomatic cases) were selected to be revisited for a study examining the long-term immune persistence of anti-HEV antibodies, as both asymptomatic and symptomatic infections are important contributors to antibody prevalence at the population level. Community health research workers attempted to revisit each of these cases at least 3 times. After written informed consent was obtained (either from the participant or from the parent or legal guardian with assent from the participant, as appropriate), a short questionnaire assessing HEV risk factors was administered, anthropometric measurements were taken, height, weight, and mid-upper arm circumference (MUAC) were measured, and 300 μL of capillary blood was drawn via fingerstick from each participant. Specimens were transferred on ice to the MHDSS laboratory within 4 hours of blood drawing and were centrifuged, and 2 aliquots of 70 μL of serum were stored at −80°C. After all specimens were collected, they were shipped in a single batch, on dry ice, to the Virology Laboratory of the ICDDR,B in Dhaka, Bangladesh. All procedures for both the initial and follow-up studies were approved by the ICDDR,B Ethical Review Committee and the Johns Hopkins Bloomberg School of Public Health Institutional Review Board.

### HEV antibody testing

Over the past decade, numerous advances have been made in the field of HEV antibody testing. The first generation of commercially available tests had poor sensitivity and specificity, often critiqued in the HEV literature (17, 29–31). An in-house assay developed by the Walter Reed Army Institute of Research (WRAIR) (Bethesda, Maryland) was considered, in the early 2000s, a “gold standard” test, and was the test used to identify asymptomatic seroconverters and symptomatic cases in the initial studies in 2003–2005 (26, 27, 31). This enzyme immunoassay (EIA) uses a truncated, recombinant HEV antigen from open reading frame 2, the capsid protein. Compared with a Western blot, this assay has 86% sensitivity and 89% specificity (31). The recommended cutoff of ≥20 WRAIR units/mL was used to classify participants as anti-HEV-positive (26, 27). The symptomatic hepatitis cases were tested with a commercially available anti-HEV immunoglobulin M enzyme immunoassay from Medical Biological Service (Milan, Italy) and the WRAIR assay described above for diagnosis of hepatitis E infection (28). These baseline tests were completed by trained virology staff at the Armed Forces Research Institute of Medical Sciences in Bangkok, Thailand, a leading reference laboratory (28).

The follow-up antibody testing was completed by trained staff at the Virology Laboratory of the ICDDR,B in Dhaka using a commercially available EIA from Beijing Wantai Pharmacy Enterprise Company Ltd. (Beijing, China), now widely considered to be among the best assays for anti-HEV detection (32, 33). This assay also employs a segment of a recombinant open reading frame 2 protein and has been validated against a number of EIAs, showing a greater degree of sensitivity (32, 34). The sensitivity and specificity of the Wantai HEV immunoglobulin G EIA are 99.5% (95% confidence interval (CI): 97.1, 100.0) and 99.6% (95% CI: 98.0, 100.0), respectively, when compared with a Western blot (35).

The performance of the Wantai assay was compared with that of the WRAIR assay in a subset of samples from the baseline seroprevalence study (2003–2005) included in this analysis (see the Web Appendix, available at https://academic.oup.com/aje). A detailed analysis of the comparability of these 2 EIAs can be found elsewhere (36). In brief, the WRAIR assay estimated the overall population seroprevalence of anti-HEV antibodies as 26.6% (95% CI: 24.0, 29.5), while the Wantai assay produced a higher estimated seroprevalence, 46.7% (95% CI: 43.5, 49.8) (*P* < 0.001), but both found about 2% seroprevalence in children under age 5 years (*P* = 1.00). The 2 assays had a 77.0% agreement (36). Using the WRAIR EIA as the “gold standard,” the Wantai EIA had a sensitivity of 94.4%, a specificity of 70.7%, a positive predictive value of 53.9%, and a negative predictive value of 97.2% (36). Therefore, the more sensitive Wantai assay was chosen for antibody testing at follow-up to minimize the risk of missing true seropositive individuals. A sensitivity analysis examining only participants whose baseline results concurred between the 2 tests is included in the Web Appendix (Web Table 1, Web Table 2).

### Statistical methods

Statistical analysis was performed using Stata, version 11 (StataCorp LLC, College Station, Texas) (37). Characteristics of persons met for the antibody persistence follow-up and those not met were compared using a rank-sum test for continuous variables and a χ^2^ test for categorical variables. After the anti-HEV testing was completed, each individual was assessed as being positive or negative for anti-HEV immunoglobulin G, based on the manufacturer’s directions, to estimate the prevalence of persistent antibodies and exact binominal 95% confidence intervals. The two groups, those with antibody persistence (seropositive at follow-up) and those who seroreverted (seronegative at follow-up), were compared using a χ^2^ test for categorical variables or a rank-sum test for continuous variables, using 2-sided tests. For asymptomatic infections, the time elapsed since exposure was calculated from the midpoint between the seronegative baseline visit and the seropositive baseline visit to the date of the follow-up visit. For participants with clinical illness, the time elapsed since exposure was calculated from the date of baseline enrollment to the date of the follow-up visit.

Reinfection with hepatitis E was assessed by asking the participants whether they had been diagnosed with hepatitis or jaundice by a health-care professional in the last 10 years. We also asked whether participants had had contact with a jaundice patient in the last 10 years (28, 38). While the accuracy of recall of these exposures may be an issue, we do not expect recall to have differed by antibody persistence status. The possibility of reexposure to HEV was assessed by asking about various water-related, sanitation, animal, and parenteral exposures. Even though contact with swine has been associated with HEV exposure (39), participants in Bangladesh were not asked about ownership of pigs, as this is a predominantly Muslim country.

Poisson regression with robust error variance was used to identify risk factors associated with antibody persistence using both univariate and multivariate methods (40). Four multivariate models were developed on the basis of both the results of the univariate analysis and the current literature. The first model included only the demographic characteristics of age and sex. The second added severity of illness (symptomatic vs. asymptomatic), which also controls for the selection of 2 distinct groups of participants. The third included all variables from the second model, adding in nutritional status. The forth included everything in model 2 plus HEV exposure characteristics of interest from the literature. This included subsequent hepatitis-like illness within the last 10 years; contact with a jaundice patient within the last 10 years; receipt of any injections within the last 10 years; type of toilet in the household; household ownership of cows, goats, or sheep; and household ownership of chickens or ducks. Model fit was assessed using the Bayesian Information Criterion (41). A sensitivity analysis of these models was performed by including only those participants for whom the WRAIR and Wantai assays agreed on antibody status in the baseline analysis (Web Table 3).

We also modeled antibody prevalence assuming that all age groups were equally likely to be infected with HEV and lost antibodies at the age-specific risks (risk ratios) observed in this follow-up study (using age at infection). The age-specific risks for antibody loss were applied to population data from Bangladesh (36), varying the percentage of the population infected with HEV. The observed and expected percentages of those anti-HEV-negative for the population for each 10-year age group were compared using a χ^2^ test.

## RESULTS

Out of the 121 potential participants, 100 (82.6%) were revisited by study staff (Table 2, Figure 1). The most common reason for loss to follow-up was permanently moving away from the study area (*n* = 12). Refusal rates were low; only 2 participants (1.7%) did not consent to participate. There were no differences in the severity of HEV infection by follow-up status.

**Table 2.** Comparison of Revisited Participants With Participants Lost to Follow-up in a Study of Hepatitis E Virus Antibody Persistence (*n* = 121), Matlab, Bangladesh, 2015

Characteristic	No. of Participants			*P* Value^a^
	Total	Revisited	Lost to Follow-up	
Mean age at follow-up, years^b^		39.7 (17.7)	44.2 (22.4)	0.404^c^
Sex				0.247^d^
Male	61	48	13	
Female	60	52	8	
Severity of HEV infection				0.615^d^
Subclinical	75	63	12	
Clinical	46	37	9	

Abbreviation: HEV, hepatitis E virus.

^a^ Two-sided *P* value.

^b^ Values are expressed as mean (standard deviation).

^c^ Rank-sum test.

^d^ χ^2^ test.

Of the 100 persons located and consenting to participate, an anti-HEV immunoglobulin G seropositivity rate of 80.0% (95% CI: 72.0, 88.0) was found, suggesting 10-year anti-HEV immunopersistence. The remaining 20% (95% CI: 12.0, 28.0) showed no detectable anti-HEV antibodies, suggesting a loss of antibodies since the time of initial infection 8–10 years before.

**Figure 2.**
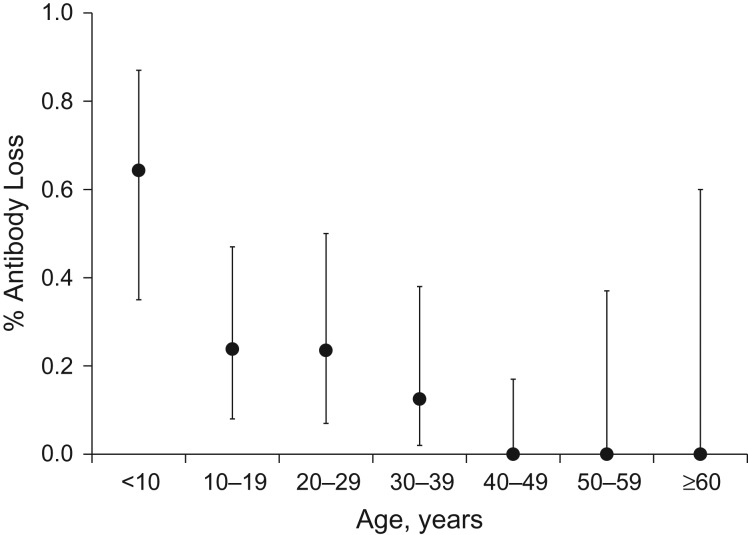
Loss of antibodies to hepatitis E virus (HEV) among persons with a previous HEV infection (*n* = 100), by age group, Matlab, Bangladesh, 2015. All 100 participants were positive for HEV antibodies at baseline (2003–2005). Sample sizes were as follows: age <10 years, *n* = 14; age 10–19 years, *n* = 21; age 20–29 years, *n* = 17; age 30–39 years, *n* = 16; age 40–49 years, *n* = 20; age 50–59 years, *n* = 8; age ≥60 years, *n* = 4. Bars, 95% confidence intervals.

Persons with persistent antibodies were generally older at infection than those who seroreverted (33.6 years vs. 14.4 years; *P* < 0.0001) (Table 3, Figure 2). There was no association between sex and antibody persistence. Among married women aged 12 years or older, an increasing number of pregnancies was associated with antibody persistence; however, older women are likely to have had more pregnancies (Table 4). Those negative at follow-up had a lower body mass index (weight (kg)/height (m)^2^) at follow-up than those who were positive at follow-up, with the difference approaching statistical significance (*P* = 0.063). MUAC did not differ between the two groups (*P* = 0.290). However, cutoffs indicating poor nutritional status for both body mass index and MUAC vary by age and do not compare well across teenagers and adults. Therefore, the percentages of participants with a low body mass index (<18.5) and a low MUAC (<22.5 cm) were calculated including only those aged ≥20 years or ≥15 years, respectively, with no differences being found using these cutoffs and age ranges. Occupation also differed by antibody persistence status (*P* = 0.014); however, this was driven by the large percentage of students among persons who were HEV-negative at follow-up (35% vs. 7.5%). All 3 participants who reported receiving a blood transfusion in the last 10 years were seropositive at follow-up, although this association was not statistically significant (Table 4).

**Table 3.** Distribution of Continuous Demographic Characteristics of Participants (*n* = 100) by Hepatitis E Virus Antibody Persistence Status, Matlab, Bangladesh, 2015

Characteristic	HEV Antibody Status at Follow-up				*P* Value^a^
	Positive (*n* = 80)		Negative (*n* = 20)		
	Mean (SD)	Range	Mean (SD)	Range	
Age at HEV infection, years	33.6 (16.7)	3.7–72.6	14.4 (10.3)	3.2–36.1	<0.0001
Time since exposure, years	1.0 (0.7)	8.78–10.7	9.5 (0.4)	8.8–10.4	0.001
Body mass index^b^	21.3 (3.3)	14.2–29.7	19.5 (3.5)	13.6–26.2	0.063
MUAC, cm	25.8 (3.0)	18.4–33.4	24.9 (2.9)	17.8–30.0	0.290

Abbreviations: HEV, hepatitis E virus; MUAC, mid-upper arm circumference; SD, standard deviation.

^a^ Two-sided *P* value (rank-sum test).

^b^ Weight (kg)/height (m)^2^.

**Table 4.** Distribution of Categorical Demographic Characteristics of Participants (*n* = 100) by Hepatitis E Virus Antibody Persistence Status, Matlab, Bangladesh, 2015

Characteristic	HEV Antibody Status at Follow-up				*P* Value^a^
	Positive (*n* = 80)		Negative (*n* = 20)		
	No. of Persons	%	No. of Persons	%	
Age at HEV infection, years					<0.0001
<10	5	6.3	9	45.0	
10–19	16	20.0	5	25.0	
20–29	13	16.3	4	20.0	
30–39	14	17.5	2	10.0	
40–49	20	25.0	0	0.0	
50–59	8	10.0	0	0.0	
≥60	4	5.0	0	0.0	
Sex					0.841
Male	38	47.5	10	50.0	
Female	42	52.5	10	50.0	
Gravidity (no. of pregnancies)^b^					0.000
0	1	2.5	4	40.0	
1–3	22	55.0	6	60.0	
>3	17	42.5	0	0	
Severity of HEV infection					0.062
Asymptomatic	54	67.5	9	45.0	
Symptomatic	26	32.5	11	55.0	
Body mass index^c^					
<18.5	16	22.2	2	81.8	0.762
≥18.5	56	77.8	9	18.2	
MUAC, cm^d^					
<22.5	9	11.4	1	6.7	0.541
≥22.5	70	88.6	15	93.3	
Occupation					0.014
Housework/none	35	43.7	9	45.0	
Farmer/fisherman/laborer	14	17.5	0	0	
Business owner	15	18.7	3	15.0	
Office-based service	5	6.3	1	5.0	
Student	6	7.5	7	35.0	
Other	5	6.3	0	0	
Type of work					0.116
Indoor	49	61.3	16	80.0	
Outdoor	31	38.7	4	20.0	
Self-reported hepatitis (last 10 years)	24	30.0	10	50.0	0.091
Contact with a jaundice patient (last 10 years)	35	43.8	10	50.0	0.615
Receiving injections (last 10 years)	49	61.3	17	85.0	0.045
Injected contraceptive (past year)^b^	6	15.0	1	10.0	0.684
Receiving blood transfusion(s)	3	3.8	0	0	0.379
Drinking water source					0.363
Tube well	66	82.5	19	95.0	
River	2	2.5	0	0	
Other	12	15.0	1	5.0	
Type of toilet					0.762
Unsanitary (open/hanging/pit)	35	43.7	8	40.0	
Sanitary (sealed/slab/flush)	45	56.3	12	60.0	
Hand-washing					
Before eating	80	100.0	20	100.0	1.000
After defecation	80	100.0	20	100.0	1.000
Eating outside the home, times/week					0.435
0 (never)	47	58.8	13	65.0	
<7	15	18.7	5	25.0	
≥7	18	22.5	2	10.0	
Animal(s) owned by household					
Cow	27	33.8	5	25.0	0.453
Goat/sheep	6	7.5	2	10.0	0.712
Chicken/duck	44	55.0	15	75.0	0.104
Rats in the home^e^	78	98.7	19	95.0	0.289

Abbreviations: HEV, hepatitis E virus; MUAC, mid-upper arm circumference.

^a^ Two-sided *P* value (χ^2^ test).

^b^ Among married women aged 12 years or older (*n* = 40 seropositive at follow-up; *n* = 10 seronegative at follow-up).

^c^ Weight (kg)/height (m)^2^. Comparison was restricted to participants aged 20 years or older (*n* = 72 seropositive at follow-up; *n* = 11 seronegative at follow-up).

^d^ Comparison was restricted to participants aged 15 years or older (*n* = 79 seropositive at follow-up; *n* = 16 seronegative at follow-up).

^e^ Seen in household in the last 30 days.

Some characteristics were associated with antibody persistence, but not in the expected direction. Elapsed time between infection and retesting was associated with antibody persistence (*P* = 0.001) (Table 3). Persons who tested positive at follow-up had a longer duration of time between the infection and retesting (mean = 9.99 years) than those who were negative at follow-up (mean = 9.45 years). However, there is only a 6-month difference between the two groups, which probably has limited practical application. Additionally, persons with an asymptomatic infection were somewhat more likely to have persistent antibodies; the difference approached statistical significance (*P* = 0.062). The participants who were seronegative at follow-up were more likely to have reported hepatitis in the last 10 years (*P* = 0.091), other than the infection used to qualify for this follow-up, and to have reported receiving an injection in the last 10 years (*P* = 0.045). These exposures, however, were based solely on participant recall over the past 10 years and therefore warrant a more detailed examination in the future. None of the other exposure characteristics examined were associated with antibody persistence status (Table 4).

In univariate Poisson regression (Table 5), only age was statistically associated with antibody persistence, with each 10-year increment of age decreasing the risk of being negative at follow-up by 51%. In the multivariate analysis, age was statistically associated with antibody status at follow-up across all models. Despite the different characteristics examined in each model, the association of age stayed relatively constant with antibody persistence, with each 10-year increment of age decreasing the risk of seroreversion by about 50%. Model 1, which only included the demographic characteristics of age and sex, had the lowest Bayesian Information Criterion, indicating the best fit among the models examined.

**Table 5.** Risk Factors for Loss of Hepatitis E Virus Antibodies After Hepatitis E Virus Infection (*n* = 100), Matlab, Bangladesh, 2015^a^

Characteristic	Univariate Analysis		Multivariate Models							
	RR	95% CI	Model 1^b^		Model 2^c^		Model 3^d^		Model 4^e^	
			RR	95% CI	RR	95% CI	RR	95% CI	RR	95% CI
Age (per 10-year increase)^f^	0.49^g^	0.37, 0.66	0.49^g^	0.37, 0.66	0.49^g^	0.37, 0.67	0.51^g^	0.37, 0.71	0.49^g^	0.36, 0.67
Female sex	0.92	0.42, 2.03	1.00	0.50, 1.99	1.01	0.51, 2.00	1.00	0.40, 2.55	1.23	0.54, 2.78
Symptomatic HEV infection	0.48	0.22, 1.05			0.83	0.41, 1.65	0.82	0.33, 2.07	0.99	0.50, 1.99
Low MUAC (<22.5 mm)	0.57	0.08, 3.89					0.73	0.15, 3.61		
Subsequent HLI (last 10 years)	1.94	0.89, 4.22							1.87	0.98, 3.58
Contact with jaundice patient (last 10 years)	1.22	0.56, 2.68							0.61	0.29, 1.25
Receiving injections (last 10 years)	2.92	0.91, 9.32							2.82	0.84, 9.40
Sanitary toilet (sealed/slab/flush)	1.13	0.51, 2.53							1.58	0.80, 3.10
Animal(s) owned by household										
Cow	0.71	0.28, 1.79							0.60	0.27, 1.32
Goat/sheep	1.28	0.36, 4.58							1.11	0.53, 2.34
Chicken/duck	2.08	0.82, 5.31							3.24^g^	1.23, 8.56

Abbreviations: BIC, Bayesian Information Criterion; CI, confidence interval; HEV, hepatitis E virus; HLI, hepatitis-like illness; MUAC, mid-upper arm circumference; RR, risk ratio.

^a^ Results were derived from univariate and multivariate Poisson regression models.

^b^ Model 1 (demographic characteristics) adjusted for age and sex (BIC = −401.0).

^c^ Model 2 (demographic characteristics + disease characteristics) adjusted for model 1 variables plus severity of disease (BIC = −396.6).

^d^ Model 3 (demographic characteristics + disease characteristics + nutritional characteristics) adjusted for model 2 variables plus MUAC (*n* = 95) (BIC = −366.3).

^e^ Model 4 (demographic characteristics + exposure characteristics) adjusted for model 2 variables plus subsequent hepatitis, contact with a jaundice patient in the last 10 years, receiving injections in the last 10 years, type of toilet in the household, household ownership of cows, goats, or sheep, and household ownership of chickens or ducks (BIC = −373.1).

^f^ All models used age (years) at HEV infection.

^g^ *P* < 0.05 (2-sided *P* value).

**Figure 3.**
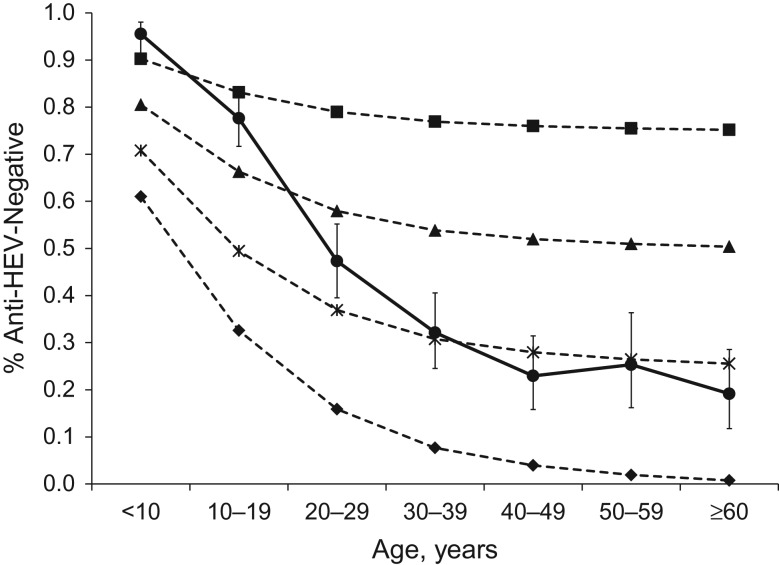
Observed and expected prevalences of negativity for hepatitis E virus (HEV) antibodies (*n* = 1,009), by age group, accounting for different risks (risk ratios) of antibody loss, Matlab, Bangladesh, 2015. Observed antibody prevalence obtained from a 2003 study in Matlab (36) is indicated with a solid line. Expected antibody prevalence (dashed lines) was calculated from univariate Poisson regression with age at infection from the current follow-up study of antibody persistence (2015), assuming varying levels of population prevalence of HEV infection. All age groups were assumed to be equally likely to be infected with HEV. ■, 25% prevalence of HEV infection; ▲, 50% prevalence of HEV infection; **X**, 75% prevalence of HEV infection; ♦, 100% prevalence of HEV infection. Bars, 95% confidence intervals.

We observed a strong positive association between young age at infection and antibody loss, both in the entire follow-up group and in the much smaller sample included in the sensitivity analysis (Table 5, Web Table 3). This indicated that persons infected with HEV at a young age were more likely to lose antibodies than those infected at an older age (Figure 2). Figure 3 displays the observed population seroprevalence in rural Bangladesh, as well as the seroprevalence at different population prevalences of HEV infection that would be expected if individuals lost antibodies at the age-specific risks (risk ratios) observed in the univariate Poisson regression of age with antibody status (Table 5). At the 75% infection percentage, the observed and expected seroprevalences did not statistically differ from each other (*P* = 0.197).

## DISCUSSION

We successfully revisited 100 persons with documented HEV infections, both asymptomatic and overt, after approximately 10 years to explore anti-HEV immunopersistence. Given the conditions in rural Bangladesh, revisiting participants after nearly a decade was difficult, as this is a highly mobile population, with addresses often lacking uniformity. However, the population of the MHDSS is extensively tracked and mapped via global positioning system technology, which contributed to the success rate of the follow-up.

The 20% rate of antibody loss in these cohorts was considerably high, given the well-documented circulation of HEV in this population. HEV is a common cause of sporadic hepatitis in Bangladesh, and frequent, seasonal flooding increases the risk of contamination of the water supply (25). Persons who were negative at follow-up were younger than those who were positive at follow-up, with younger age at infection increasing the risk of seronegativity at follow-up in both univariate and multivariate analyses, as well as in the sensitivity analysis. The association of seroreversion with younger age at infection may provide insight into the unique epidemiology of HEV in South Asia.

The epidemiology of HEV differs from that of other enterically transmitted infections, with perplexingly low rates of antibody prevalence in young children (5). The most common explanation, to date, is that children are somehow less likely to be infected with HEV, possibly through less exposure, or that the immune system does not recognize the pathogen in the same manner in children as in adults (4, 42). Our results, however, suggest that an infection with HEV at a young age, whether asymptomatic or symptomatic, is more likely to lead to a transient antibody response than an infection at an older age. We modeled population seroprevalence assuming age-dependent antibody loss after infection as the major driving factor behind the single–time-point patterns, assuming constant prevalence of HEV infection across age groups. At the 75% infection prevalence, observed seroprevalence data did not statistically differ from those predicted by the models developed from this follow-up study. These results suggest that age-dependent antibody loss may contribute to population seroprevalence, providing a possible contributing factor for the single–time-point population seroprevalence data observed across South Asia. However, more studies are needed.

In the current follow-up, we were not able to follow participants very closely over time, and this probably contributed to significant misclassification—specifically in relation to time-varying and recall-based exposures, particularly a subsequent clinical HEV infection. While both asymptomatic and symptomatic reinfections with HEV have been documented (43), we were not able to examine this in detail, basing our assessment of a subsequent illness solely on participant recall. Here, we found that another instance of self-reported hepatitis illness was somewhat more likely among persons who were negative for antibodies at follow-up, possibly suggesting that certain individuals are less likely to produce long-lasting antibodies than others. However, future studies are needed to further assess the role of reinfection in antibody persistence.

A major issue with HEV research over the past 2 decades has been the lack of a highly sensitive and specific assay. Here, we used different assays to detect HEV antibodies at baseline and follow-up. Additionally, we could not be sure that the symptomatic cases were seronegative prior to their clinical HEV infection. Both of these issues could influence the overall antibody persistence seen in this study, as well as complicate the interpretation of time since exposure as a risk factor of interest. Future studies are needed to closely examine long-term antibody kinetics using consistent assays.

To our knowledge, this was the first long-term follow-up of participants with HEV infection, based on a longitudinally compiled population cohort. We were able to revisit a range of participants, reflecting the possible ways in which HEV infections are handled by the host, from very sick individuals to asymptomatic seroconverters. Globally, there is a paucity of confirmed seropositive specimens linked to known geographic location and uniquely identified patients, due not only to highly variable assays and controversies over the validity of the immunoassays available for specimen testing but also to the challenging environments in which HEV has often been studied (30, 31, 44). Not only was the initial study conducted in a setting where individuals could be followed over time, but the specimens collected in this study were tested using highly sensitive, specific, and reliable assays (35). The results of this study could provide new insight into the immunological management of HEV infection, highlighting the importance of age at infection in the ability to produce long-lasting antibodies. This finding adds a layer of complexity to the well-documented phenomenon of low rates of pediatric infection and illness for both sporadic HEV transmission and transmission during outbreaks. However, further studies are needed, as it is unclear whether this differential immunopersistence associated with age at infection reflects an immaturity of the immune response to this specific pathogen, differential doses of infective pathogen, or other confounding host factors not captured in this initial study.

## Supplementary Material

Web Material

## Abbreviations

CI: confidence interval
EIA: enzyme immunoassay
HEV: hepatitis E virus
ICDDR,B: International Center for Diarrhoeal Disease Research, Bangladesh
MHDSS: Matlab Health and Demographic Surveillance System
MUAC: mid-upper arm circumference
WRAIR: Walter Reed Army Institute of Research.
