# Supplementary material for: A 10-Year Immunopersistence Study of Hepatitis E Antibodies in Rural Bangladesh
Source: Am J Epidemiol. 2018 Mar 23;187(7):1501–10. doi: 10.1093/aje/kwy044 (PMC7113636; doi:10.1093/aje/kwy044)
Supplement: Web Material [file kwy044kmushwebmaterialfinal.pdf]

WEB APPENDIX

The performance of the Wantai and WRAIR assays were compared in a previously published paper.[1] A subset of the individuals included in that analysis were also included in this antibody persistence study. Web Tables 1 and 2 display the comparison between the Wantai and WRAIR assays for the 63 asymptomatic seroconverters (Figure 1) who had their baseline serum tested using both assays. The specimens that tested positive using the WRIAR assay were also very likely to test positive with the Wantai assay. However, many of the specimens that were negative by WRIAR were found positive by Wantai.

A sensitivity analysis was performed by recreating the regression models only including the participants where the WRAIR and Wantai assays agreed on the antibody status in the baseline analysis (n = 48) (Web Table 3). Severity of infection and Model 2 (demographic characteristics plus severity of infection) were not included in the sensitivity analysis as all participants whose baseline samples were analyzed with both tests experienced an asymptomatic infection. Here, younger age was statistically associated with antibody loss across all models, both univariate and multivariate, with a similar order of magnitude compared to the entire antibody persistence follow-up. As with models including all the participants in the follow-up, Model 1 (demographic characteristics of age and sex) had the lowest BIC, indicating the best fit of all the models examined. In the univariate analysis, low MUAC and owning goats or sheep decreased the risk of seroreversion at follow-up. In Model 4 (demographic and exposure characteristics), self-reported injections in the last 10 years increased the risk of seroreversions while older age, owning cows, and owning goats or sheep decreased the risk of

seroreversion. However, due to the small sample size in the sensitivity analysis, some categories did not have any individuals, making the estimates very unstable, particularly in the more complex models.

A major issue with HEV research over the past two decades has been the lack of a highly sensitive and specific assay. The WRAIR test, used to identify baseline cases in this study, while considered a gold standard test at the time of the baseline analysis, has since been shown to lack sensitivity, missing nearly half of HEV-positive individuals compared with the Wantai assay.[1] We found that those WRAIR identified as positive were likely to be found positive by the Wantai assay as well. However, the WRAIR assay likely missed many HEV positive individuals at baseline. In this study, those identified as asymptomatic seroconverters in the baseline study may not have seroconverted during the time tested, but at some point in the past. This issue complicates using time since exposure as a risk factor of interest in this analysis, as the negative samples may not have truly been negative. However, the individuals examined in this study were still infected with HEV, but the exact timing of the exposure is difficult to analyze due to the use of the WRIAR assay at baseline. A few more exposure characteristics were found to be associated with antibody persistence status in the sensitivity analysis, likely due to the small sample size classifying few or no individuals into some categories leading to highly unstable estimates.

Web Table 1. Comparison of 2 Anti-HEV EIAs from Banked Sera in Participants in Matlab, Bangladesh, 2004–2005 (*n* = 63)

		**WRAIR Total IgG**		
		**Positive**	**Negative**	**Total**
Wantai IgG	Positive	35	10	45
	Negative	5	13	18
	Total	40	23	63

EIA, enzyme immunoassay; HEV, hepatitis E virus; Ig, immunoglobulin; WRAIR, Walter Reed Army Institute of Research.

Web Table 2. Wantai IgG and WRAIR Total Ig Comparative Test Performance From Banked Sera in Participants in Matlab, Bangladesh, Using the WRAIR Test as the "Gold Standard," 2004–2005 (n = 63)

Characteristic	Estimate (%)	95% CI (%)
Sensitivity	87.5	73.2, 95.8
Specificity	56.5	34.5, 76.8
Positive predictive value	77.8	62.9, 88.8
Negative predictive value	72.2	46.5, 90.3
% agreement	76.2	63.8, 86.0

CI, confidence interval; Ig, immunoglobulin; WRAIR, Walter Reed Army Institute of Research.

Web Table 3. Results of Univariate and Multivariate Poisson Regression Models for Risk Factors for Antibody Loss After HEV Infection, Matlab, Bangladesh, 2015 (WRAIR and Wantai Agreement at Baseline Cohort n = 48)

	Univariate Analysis		Multivariate Models					
			Model 1[a]		Model 3[c]		Model 4[d]	
	**RR**	**95% CI**	**RR**	**95% CI**	**RR**	**95% CI**	**RR**	**95% CI**
Age (per 10-year increase)[e]	0.48[f]	0.31, 0.73	0.49[f]	0.31, 0.76	0.49[f]	0.30, 0.80	0.45[f]	0.27, 0.75
Female sex	0.54	0.13, 2.17	0.74	0.20, 2.80	0.69	0.14, 3.27	0.46	0.09, 2.40
Symptomatic infection	NA							
Low MUAC (<22.5 mm)	2×10^{-7} [f]	9×10^{-8}, 9×10^{-7}			6×10^{-7} [f]	8×10^{-8}, 4×10^{-6}		
Subsequent HLI (last 10 years)	1.52	0.34, 6.81					0.86	0.21, 3.55
Contact with jaundice patient (last 10 years)	1.05	0.26, 4.25					0.38	0.06, 2.22
Injection (last 10 years)	4.29	0.55, 33.6					14.6[f]	1.99, 107.1
Sanitary toilet	1.13	0.28, 4.57					0.31	0.05, 1.80
Household ownership								
Cow	0.38	0.05, 2.84					0.19[f]	0.04, 0.91
Goat/sheep	8×10^{-7} [f]	2×10^{-7}, 4×10^{-6}					3×10^{-6} [f]	2×10^{-7}, 5×10^{-5}
Chicken or duck	0.73	0.18, 2.93					0.75	0.15, 3.66

CI, confidence interval; HLI, hepatitis-like illness; HEV, hepatitis E virus; MUAC, mid-upper arm circumference; RR, risk ratio; WRAIR, Walter Reed Army Institute of Research.

P values < 0.05 were considered significant.

[a] Model 1 (demographic characteristics) is adjusted for age and sex. Bayesian Information Criterion: −156.5765.

[c] Model 3 (demographic + nutritional characteristics) is adjusted for model 1 plus mid-upper arm circumference (MUAC). Bayesian Information Criterion: −140.4475.

[d] Model 4 (demographic + exposure characteristics) is adjusted for model 1 plus subsequent hepatitis, contact with a jaundice patient in the last 10 years, injections in the last ten years, type of toilet and household ownership of cows, goats or sheep, and chickens or ducks. Bayesian Information Criterion: −135.179.

[e] All models use age at infection.

[f] RR with a two-sided P value < 0.05.

Web Reference

1. Kmush BL, Labrique AB, Dalton HR, et al. Two generations of "gold standards": the impact of a decade in hepatitis E virus testing innovation on population seroprevalence. *Am J Trop Med Hyg*. 2015;93(4):714–717.
